# Global distance learning for Japanese generalists: The new era of learning

**DOI:** 10.1002/jgf2.275

**Published:** 2019-09-09

**Authors:** Takashi Watari

**Affiliations:** ^1^ Postgraduate Clinical Training Center Shimane University Hospital Shimane Japan

To the Editor

I have completed the Global Clinical Research Training Course (and Introduction to Clinical Research Training Course) from Harvard Medical School. The course duration was from December 2017 to June 2019. I would like to recommend this course because I believe that this course is a very effective and efficient way for young generalists in Japan to learn how to conduct clinical research.

Since ancient times, the significant role of university hospitals in the three pillars—clinical approach, education, and research—has remained the same. However, because of my experience as faculty staff in a university hospital, I feel that for many generalists who work at universities, the most unstable pillar of the three is “research.” This has also been reported in previous studies.^1^, ^2^ However, there are limited opportunities to avail research support and mentoring for generalists in Japan.^3^ I believe that the reasons are as follows. First of all, the research system in Japan (mainly experimental basic medicine) has progressed based on organ‐specific medicine. Hence, there is a dissociation among the generalists’ interest, which is predominantly from a cross‐sectional and bird's‐eye perspective for the patients and community health, and the methodology which is based on experimental medicine. Second, it is difficult to obtain a good research mentor to conduct clinical research in a clinical setting, as the medium‐size facilities lack sufficient research infrastructure.

Therefore, how can we foster the growth of a generalist who acquires research skills and plays an active role in these pillars of research? I am convinced that global distance learning education is a solution. Harvard Medical School GCSRT is an Internet‐based distance education portal, where over 150 students from over 65 countries gather to study clinical research together.^4^ Many practical classes are available for busy clinical practitioners to take at their convenience. Compulsory classes like the “Interactive Webinar” are held for students from all over the globe, between 21:00 and 24:00 in Japan standard time, and it is possible to participate if we manage our respective time schedules. In addition, the unique content comprises designing a clinical research plan, the method of actual testing using the statistical software Stata, and the skill of drafting a research plan to apply for international research grants. I would like to emphasize that the course content concentrates on very practical aspects.

I believe that a generalist who gets to see the patient is likely to raise important clinical research questions. In that sense, I am convinced that global distance education is a new learning method through which practicing generalists in the community can keep themselves updated and learn suitable sophisticated skills.

## CONFLICT OF INTEREST

The authors have stated explicitly that there are no conflicts of interest in connection with this article.

## AUTHOR CONTRIBUTIONS

The author had access to the information used, and the author participated in the preparation of this manuscript.
